# Effects of soil amendments on growth and biomass yield of early generation seeds of sweet potato (*Ipomoea batatas* (L.) Lam) grown in net tunnels

**DOI:** 10.1371/journal.pone.0290585

**Published:** 2023-11-15

**Authors:** Daniel Markos, Fekadu Gurmu

**Affiliations:** 1 Southern Agriculture Research Institute, Crop Sciences Research Directorate, Hawassa, Ethiopia; 2 Ethiopian Institute of Agricultural Research, Crop Sciences Research Directorate, Addis Ababa, Ethiopia; Universidade Federal de Minas Gerais, BRAZIL

## Abstract

Early generation sweet potato vines are multiplied on nursery beds with high densities using soil medium within insect-proof net tunnels to inhibit the entrance of virus-transmitting insects (aphids and white flies). However, the rapid multiplication beds require suitable soil amendments to support vigorous growth of vines. To this end, farmyard manure, wood ash, sawdust, compost, coffee husk and control (soil only) were evaluated using Randomized Complete Block Design with three replications during the belg and meher seasons of 2016 to 2017 in the Chefe testing site of Hawassa Agriculture Research Center. Results also showed that the soil pH was higher by 8.9% and 1.4% due to the application of wood ash and sawdust whereas there was 9.3, 5.9 and 0.9% decrease in soil pH due to the use of compost, coffee husk and farmyard manure, respectively. All soil amendments enhanced soil moisture conservation compared to the control. A 47.0%, 31.2%, 30.3% and 26.5% increase in cation exchange capacity was observed by the end of second year due to use of coffee husk, wood ash, sawdust and farmyard manure, respectively. The potassium content was increased by 47.0%, 45.5% and 35.9% due to the use of wood ash, farmyard manure and coffee husk, respectively. The pencil root length, pencil root width and below-ground biomass were not affected significantly (P < 0.05) by differences in soil amendments (P < 0.05) in this experiment. The percentage increment in above-ground biomass due to the farmyard manure, coffee husk and compost was 62.01%, 53.56% and 49.87%, respectively higher than the control. Sawdust suppressed vine growth compared to the control. Coffee husk and farmyard manure medium produced higher number of branches (23 branches / plant) during meher whereas coffee husk produced the same during belg (14.4 branches / plant). Significantly (P < 0.05) higher number of vines was obtained due to farmyard manure (4686 vines/m^2^) and coffee husk (4602 vines/m^2^) compared to the control (2683 vines/m^2^). Significantly longer internodes were recorded due to the farmyard (100.6% greater) compared to the control. Thus, 50% farmyard manure, 50% coffee husk or 50% compost are recommended for better growth of pre-basic sweet potato vines in net tunnels. This study proclaimed possibility of nursery bed vine multiplication through use of local organic residues at the level of small holders and research sites during short and long rain seasons.

## 1. Introduction

Sweet potato ***(****Ipomia batatas (L*.*)* Lam**)** is grown in over one hundred countries in tropical, sub-tropical and temperate climates. The crop has total annual production of 106–110 million tons in an area of 8.1 million hectares [1,2]. It is a staple food for many people in South-East Asia, Latin America and Africa (including Ethiopia) who produce the plant from its vines, roots or tissue cultured plantlets. However, owing to changing climate and food habits, growing demand for sweet potato planting materials has been reported in Ethiopia [3]. Sweet potatoes produced in tissue culture laboratories are generally small miniature plantlets with weak root and shoot systems. These are hardened off in screen houses and net tunnels to adapt harsh environments prevailing in the field. These materials are called early-generation seed (pre-basic). Thereafter, sweet potatoes are propagated using vine cuttings obtained either from freshly harvested plants or nursery. However, repeated use of vines collected from same field can cause increased weevil infestation and subsequent yield reductions. Vines obtained from nurseries should be healthy and vigorous for maximum root production. Healthy cuttings of sweet potatoes respond very well to soil amendments and applied fertilizers under growing conditions [4,5]. The threat of sweet potato health has been associated to sweet potato feathery and mottled viruses that are the two chronic diseases which resulted in total sweet potatoes failure in southern Ethiopia during 2010 to 2014 growing seasons [6]. The vectors for these diseases were white flies and aphids, which necessitated multiplication of early-generation seeds in net tunnels. Moreover, there were complex signs and symptoms of nutritional disorders observed among growing vines. However, sweet potato vine business in Ethiopia is dominated by the informal sector; demand for quality planting materials of improved varieties is low and inconsistent. This was mainly due to lack of demand for quality declared seed (QDS from root producers, which was in turn due to lack of awareness, low willingness to pay, and lack of registered QDS producers) [6]. Therefore, there was a need to multiply large amounts of plantlets in Eastern and Southern Africa in order to respond for ever increasing demand of insect pest and disease free sweet potato planting materials required in a very short period of time. The fact that growers have limited access to timely availability of quality sweet potato vines also contributed to sub-optimal root yields [7].

Depending on the growing conditions, vines are harvested from 80^th^ to 100^th^ days after planting or last cutting [4]. Increasing vine production per unit area and per unit time could be obtained through increasing plant population, growing in high rainfall and temperature conditions, and using nitrogenous fertilizers [8,9]. Irrigating twice a day (early morning and late afternoon) has been practiced during non-rainy times [5]. Organic amendments are bulky, have low nutrient concentrations that are released slowly and do give lower yield when used alone [10]. However, soil amendments with organic manure improve physical properties, microbial activity and nutrient availability there by boosting organic production [11]. Sas-Paszt *et al*. [12] compared different mulching materials and elaborated that only sawdust mulch significantly increased the number of apple fruit in size diameter class of 7.0–7.5 cm compared with the control. They also noted that the best pH and organic matter results were observed with the use of the compost, cow manure and the *mycorrhizal* substrate, whereas the concentrations of P, K and Mg, most of micronutrients and soil organic matter were also elevated. However, crops like sweet potato rarely benefit from fertilizer application mainly because they were branded wrongly poor *man’s crop* requiring little or no external nutrients. The bulkiness of organic amendment can be reduced through proper management during preparation, application, storage, handling and enrichment, and this helps to reduce drudgery. Soil macro- and micronutrient mining and organic matter depletion could also result from an extensive farming system with less nutrient replenishment, crop residue removal, inefficiency of soil nutrient input, excessive tillage, over grazing, deforestation, and soil erosion. Hence, there is a need to enhance soil physical and chemical conditions in the net tunnels used for sweet potato multiplication. Thus, this experiment was designed with objective of identifying suitable soil amendments that enhance soil physic-chemical conditions and maximize vine production of sweet potatoes in net tunnels.

## 2. Materials and methods

### 2.1. Description of the study area

The study was carried out at Hawassa Agricultural Research Center Hawassa Chefe Kote Jebesa research Station (Altitude 1700 m a.s.l, 07°03’51.8” N latitude and 038°28’52.2” E longitude) inside net tunnels used for early generation seed multiplication from 2016 to 2017. The area receives a total of 1000 to 1200 mm rainfall in bimodal raining pattern with short rains (also called belg rains) coming from April to May where as long rains (also called meher rains) coming from July to October. The mean annual minimum and maximum temperatures of the area were 16.5°C and 29.2°C, respectively.

### 2.2. Experimental design and field management

The experiment consisted of six treatments arranged in a Randomized Complete Block Design with three replications both in *belg* and *meher* seasons. The treatments included 50% farmyard manure (10 kg/m^2^), 50% saw dust (10 kg/m^2^), 50% wood ash (10 kg/m^2^), 50% compost (10 kg/m^2^), 50% coffee husk (10 kg/m^2^) and 100% soil medium. Wood ash was brought from villages and farmyard manure was obtained at 50% moisture from dairy farms at Hawassa. Compost was prepared following heap composting procedures. Coffee husk was obtained from coffee pulpery in *Yirgalem* area. The saw dust was obtained from Hawassa chip wood factory. As all of these materials are by-products, payments were done only for labour during collection. The composition of soil medium would be half and that of added medium would be half (50:50 m/m (mass basis) in all treatments except the control and mixed well with the soil through tillage operation. Uniform depth of each treatment was established in every bed by mixing the medium uniformly with the top soil. Each treatment was laid in a plot size of 2 m x 1 m in a raised bed, and plants were spaced by 20 cm x 10 cm row by plant spacing, respectively. The test sweet potato variety called Kulfo (LO3232) was grown in all the beds on the same date. 50 g of urea was top dressed uniformly in each rapid multiplication bed one month after planting. For *belg* season cropping, the soil amendments were incorporated to the soil in the first week of February and the vines were planted in the first week of March every year. For *meher* planting, soil amendments were incorporated in June and planting of vines was carried out in July. The crop was hand-weeded weekly from the second week until the sixth week until weed invasion was less problematic due to leaf canopy closure. Harvesting was done in the fourth month.

### 2.3. Data collection

#### 2.3.1. Plant sampling and measurements

**Fig 2:**
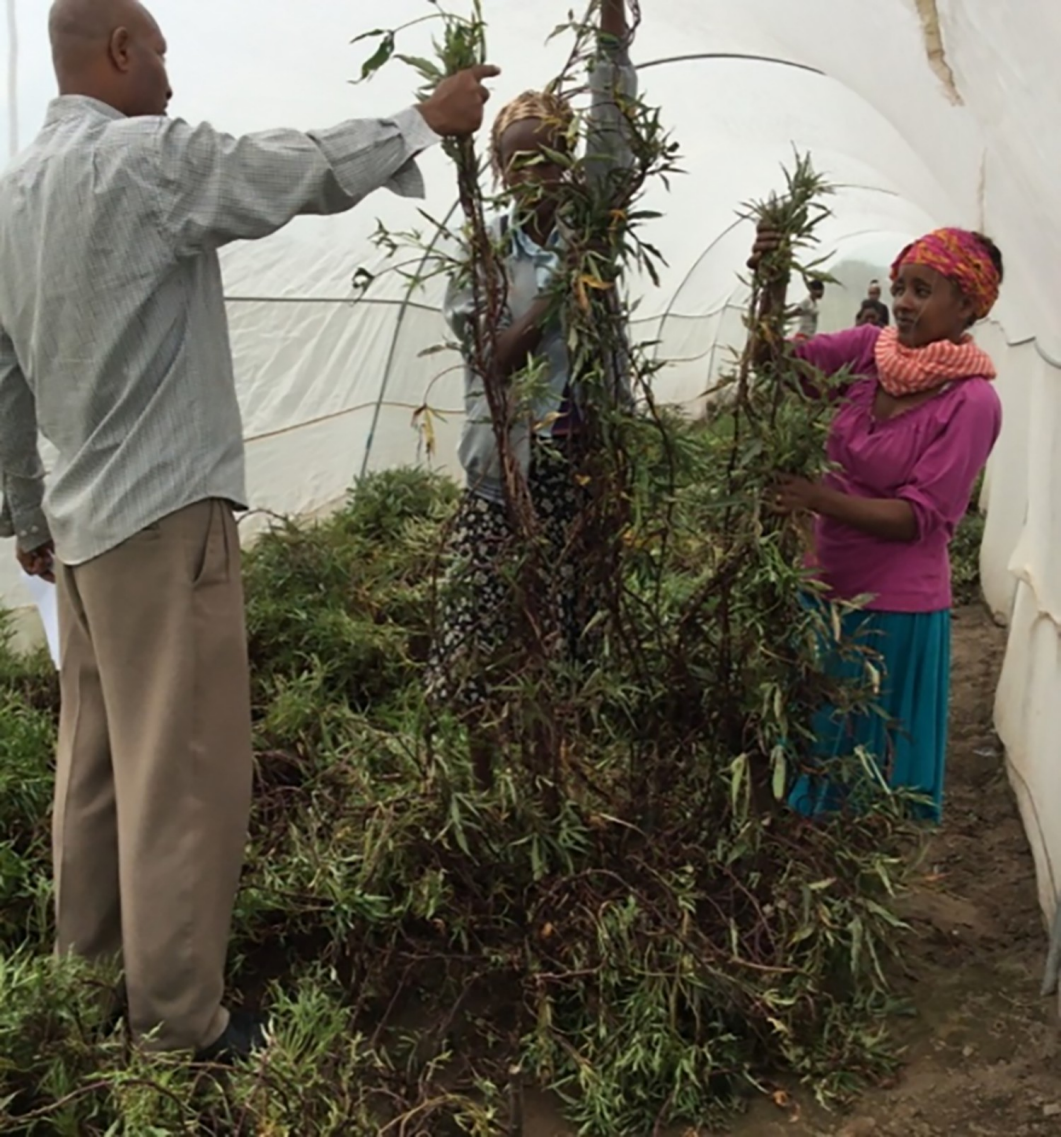
Vine measurment using mesasuring tape inside net tunnel.

Plants were selected at random from the center row (datum row) and two plants at each end of the center row were considered buffers. Vine length/branch, branch number/plant, and leaf number/plant were measured in the second, third, and fourth month of planting. Branch number/plant was counted from randomly selected five plants and divided by number of sampled plants to get branch number/plant. Leaf number per plant was counted randomly from five plants grown in datum rows of a plot (Fig 2). Because a vine should have length of 30 cm, this magnitude was considered in the Eq 1. The vine length was measured from twenty branches that were sampled randomly. Subsequently, number of vines per plant and number of vines/ha were calculated as given in Eqs 1 and 2:

$$Number\;of\;vines\;per\;plant=\frac{(Average\;number\;of\;branches\;per\;plant)*{\left(Average\;length\;of\;vines\;per\;branch\right)}^{\;}}{30\;cm}.$$ Eq 1

$$Vines\;per\;hectare=(Average\;number\;ofvines\;per\;plant)*{\left(Average\;number\;of\;plants\;per\;hectare\right)}^{\;}$$ Eq 2

The number of internodes/plants was counted from randomly selected five plants and divided by number of sampled plants to get the number of internodes/plant. The length of internodes was measured from twenty vines that were sampled randomly and divided by the number of sampled vines to get the mean length of internodes/plant. The yield of above-ground biomass (Byld) was measured by weighing all above ground vegetative parts of plants found in datum rows by the end of the fourth month during harvesting. The yield of below-ground biomass (Pryld) was measured by weighing pencil roots and adventitious roots after harvesting plants found in datum rows by the end of the fourth month during harvesting. Pencil root length (PRL) and pencil root width (PRW), which are dimensions of miniature thin storage roots produced in nursery beds during three to four month period. The roots were measured from randomly selected ten pencil roots harvested from the net plot area. The change in vine yield or other measured trait has been given as shown in Eq 3 below.


$${\%\;Change\;in\;vine\;yield\;}^{\;}=\frac{(Vine\;yield\;due\;to\;treatment-Vine\;yield\;due\;to\;control)*100}{Vine\;yield\;due\;to\;control}+\ldots .$$ Eq 3

#### 2.3.2. Soil sampling and measurements

Regarding soil physico-chemical properties, electrical conductivity was measured using ES ISO 11265: 2014 procedures, and pH-H_2_O was measured through ES ISO 10390: 2014 procedures. The percentage of sand, clay and silt was analyzed using Boycous Hydrometer, and texture was determined using a textural triangle. Total nitrogen was determined using ES ISO 11261:2015 procedures. CEC was analyzed using the ammonium acetate method. B, Ca, Co, Fe, K, Mg, Mn, Na, P, S, Zn, Al, Mo, and Si were determined using Mehlich-III procedures in Horticoop Ethiopia soil and water analysis laboratory in Debrezeit, Ethiopia [13].

### 2.4. Data analysis

Prior carrying out analysis of variance, homogeneity test was done using Bartlett’s test for years [14]. Subsequently, a two way ANOVA (treatment x season) was used to analyze the response of growth and vine components in the experiment. The SAS 9 computer software was used. A post hoc F test was done using LSD to separate treatment means at a probability level of 5% and 1% whenever treatments were significant.

### 2.5 Full ethics statement

Regarding the permits of the work, this research has been carried out in SARI (Southern Agricultural Research Institute), which is public institution established to carry out public research, generate information and to transform the agriculture sector in the country. The current study does not have possible impact on endangered or protected species, and does not involve human interviews. Moreover, the methods, data and results were presented transparently.

## 3. Result and discussion

### 3.1 Soil physical and chemical properties

The soils of the study area are sandy loam in texture. The macro-nutrients (N, P, K, Ca, Mg) and micronutrients (Fe, Zn, Na, B, Cu, Fe, Si, Al, Mn, Mo and Co) contents were also analyzed and reported. Results of soil analysis after vine harvesting showed that Fe, Zn, Na, B, Cu, Fe, Si and pH increased significantly due to 50% wood ash treatment compared to other soil mediums (Annex table 3 and 4 in S1 File). Iron (III), manganese (II), zinc (II), copper (III) and iron (III) were in sufficient rating [14–23]. Sawdust produced significantly higher amounts of Ca, N, and soil moisture compared to other mediums used to grow sweet potatoes. Coffee husk produced higher CEC compared to other soil amendments. Since CEC measures the extent how soils can hold cations by negatively charged colloids onto essential nutrients and minerals with the fact that higher CEC showing more availability of nutrients to plant roots that need them for survival and functioning. Farmyard manure resulted in high CEC, K, and S but intermediate Zn content of the media. Compost resulted in low pH, CEC, and K but intermediate N, P and Ca (Annex table 3 and 4 in S1 File). Nitrogen content was very low due to soil medium and wood ash, low due to farmyard manure, compost and coffee husk but optimum for saw dust [20]. When pH was considered, there was an 8.9% and 1.4% increment in pH due to the application of wood ash and sawdust whereas there was a 9.3, 5.9 and 0.9 decrease in pH due to the use of compost, coffee husk and farmyard manure. pH due to farmyard manure, saw dust and soil medium were moderately alkaline whereas due to compost and coffee husk were neutral [20]. Thus, organic residues used in the study prevented low pH and subsequently, low vine yield which would otherwise have caused weak root growth, which in turn was associated with H+ ion injury, toxicities of aluminum (Al) or manganese (Mn) and deficiencies of calcium (Ca), phosphorus (P) or magnesium (Mg). This agreed with findings of Sas-Paszt *et al*. [12] who reported the best pH and organic matter results from the use of the compost, cow manure and the *mycorrhizal* substrate, whereas the concentrations of P, K, Mg, most of micronutrients and soil organic matter were also elevated due to the use of organic resources. Results of these study were in line with Bedada *et al*. [24] who reported a 0.83 mg/kg B, 251 mg/kg Mn, 2.41 mg/kg Cu, 143 mg/kg Fe and 18.1 mg/kg Zn for compost used in *Beseka* area of Ethiopia. Our report also confirmed that the micronutrient concentrations were greater in soil amended plots compared to the untreated plots.

All soil amendments contributed to moisture conservation compared to soil medium. The use of each of these soil amendments improved the N content compared to the soil medium. Sawdust produced high N; coffee husk, compost and farmyard manure produced medium N, and wood ash was the low in N content [20]. The use of wood ash and farmyard manure medium increased the P content by a 43% and 33.4% (Annex table 4 in S1 File). Application of coffee husk, farmyard manure and wood ash increased the EC content by a 169.2%, 146.2% and 76.9% respectively compared to the soil medium; however, there was a 23.1% decrease in EC due to the use of saw dust in the soil amendments. A 47.0%, 31.2%, 30.3% and 26.5% increase in CEC was observed by the end of the second year due to the use of coffee husk, wood ash, sawdust and farmyard manure, respectively. The K content was increased by 47.0%, 45.5% and 35.9% due to use of wood ash, farmyard manure and coffee husk medium, respectively (Annex table 5 in S1 File). Phosphorus was very high across all medium. The CEC content optimum to high but K content is very high [Annex table 6 in S1 File]. The current result is on par with the finding of Kundu *et al*. [25] who reported more moisture, enhanced mineral N (29–87%), P (1.4–12.6%) and K (16–36%) availability when applied for dry season sweet potato production.

### 3.2. Mean squares of sweet potato growth and vine yield components

**Fig 1:**
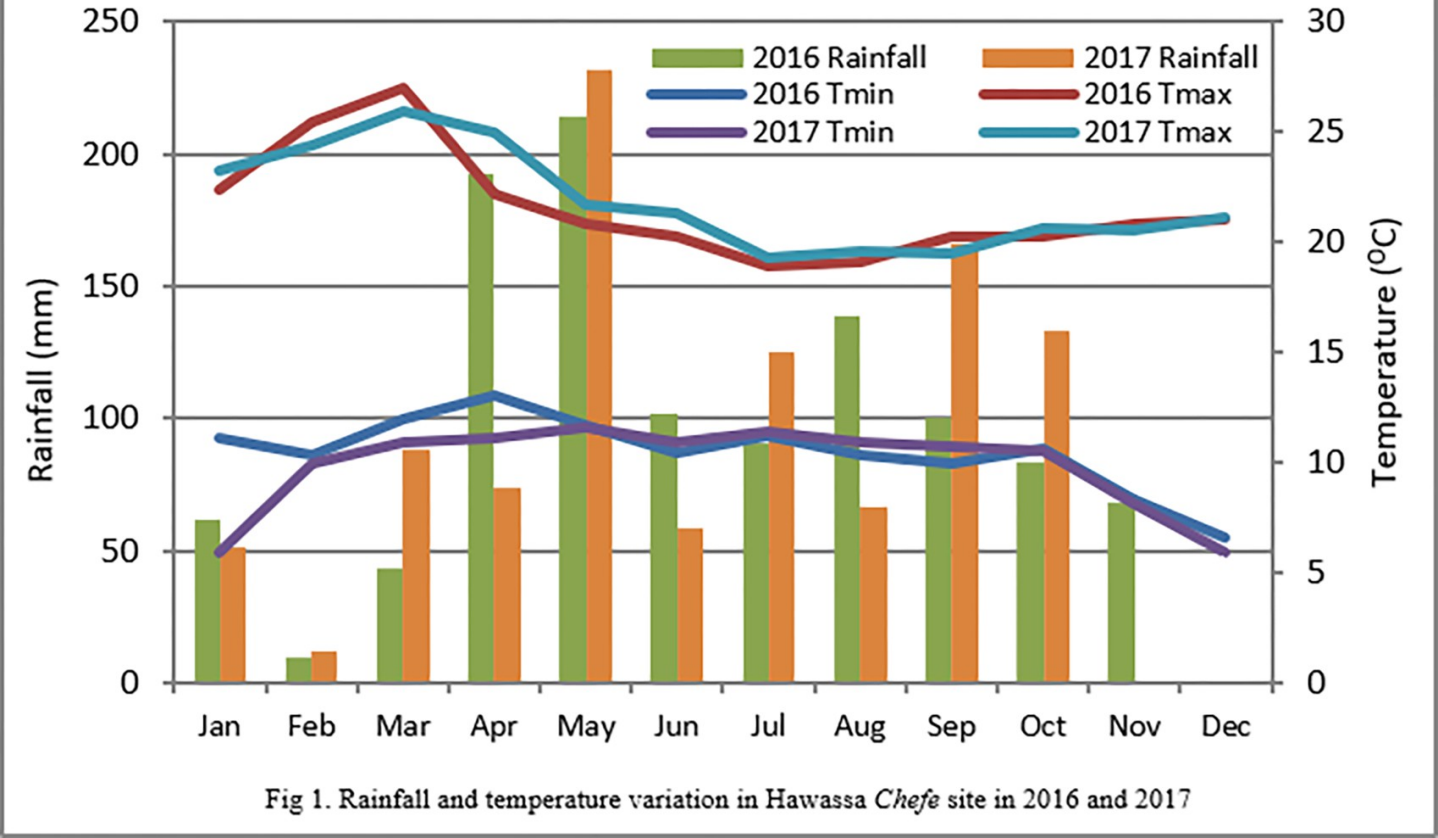
Rainfall and temperature variation in Hawassa *Chefe* site in 2016 and 2017.

Because variances across years were homogenous in Bartlett’s Test [14], combined analysis was performed in this analysis. Analysis of variance results showed a significant (P < 0.05) difference in vine length and leaf number measured after 2^nd^, 3^rd^ and 4^th^ months from planting due to effect of season. Only after 4^th^ month of sweet potato plating did branch number show this trend. The difference in seasons also caused significantly (P<0.05) effect on the pencil root length, pencil root width, above and below—ground biomass of sweet potato in the study area. The effect of the season was strong and significant on most growth and biomass components (Annex table 1 and 2 in S1 File). This could be due to variations in rainfall during the establishment and vegetative growth stages of the plant (Fig 1). In fact, there were insignificant (P<0.05) variations in minimum, maximum and average temperatures in 2016 and 2017 in the study site (Fig 1). However, the effects of the season was not significant (P<0.05) on branch number measured after 2^nd^ and 3^rd^ month of sweet potato planting, number and length of internodes during the study period (Annex table 1 and 2 in S1 File).

### 3.3. Effects of season on growth and vine yield of sweet potatoes

Leaf number measured after 3^rd^ and 4^th^ month of sweet potato planting was significantly (P<0.05) greater in *belg* than *meher* season. Similarly, vine length measured after 2^nd^ and 3^rd^ month of sweet potato planting were greater in *belg* than *meher* season. However, vine length measured after 4^th^ month of sweet potato planting, branch number measured after 2^nd^ and 3^rd^ month of sweet potato planting, pencil root length and width, yield of above and below-ground biomasses were significantly (P<0.05) higher when the crop is grown in *meher* than *belg* season (Table 1). Thus, plants grown in the *meher* season had more branches (14.74 to 24.2%), had lesser leaf number, longer and wider pencil roots besides higher above-ground and below-ground biomass compared to plants grown in the *belg* season. This could be because the *meher* season marks long rains compared to the *belg* season which corresponds to short rains in the area (Fig 1). Thus, vine production would result in 50.87% more yields in the *meher* season compared to the *belg* season. However, the positive increment of vine length/plant (15.46%) and leaf number/plant (58.9%) in the *belg* season with 26, 555 kg/ha above ground biomass production showed that a sufficient amount of vines could be produced using the *belg* rains by smallholder farmers that produce sweet potato roots in the *meher* season. Small vine production at household level particularly during short rains of *belg* season could be considered a vital practice because it marks time and distance isolation from root production, a practice which is very important to restrict spread of insect pests and diseases through long distance vine transport. Those farmers that produce vines in the *belg* season do produce seeds before the main season, and address their own food security demand in succeeding season better than those who don’t have vines at their disposal.

**Table 1:** Growth and vine yield of sweet potatoes as affected by seasons.

Variables	*Belg*	*Meher*	% change	LSD
VL2M (cm)	73.5	62.1	15.5	6.75*
VL3M (cm)	100.9	87.6	13.2	5.75**
VL4M (cm)	112.3	126.6	-11.3	9.73*
B2M	8.0	9.9	-19.2	NS
B3M	13.3	15.6	-14.7	NS
B4M	15.0	19.8	-24.2	1.71**
L2M	67.3	27.6	58.9	17.7*
L3M	125.6	36.0	71.3	16.7**
L4M	122.8	42.4	65.5	9.8**
PRL (cm)	7.5	12.1	-38.0	2.75*
PRW (cm)	3.4	21.1	-83. 9	2.54**
Byld (kg/ha)	26555	54055	-50.9	10605**
Pryld (kg/ha)	3961.1	7044.4	-43.8	1345*

VL2M- vine length after two months, VL3M-vine length after three months, VL4M-vine length after four months, B2M –branch number after two months, B3M - branch number after three months, B4M - branch number after four months, L2M –leaf number after two months, L3M –leaf number after three months, L4M-leaf number after four months, Byld–yield of above-ground biomass, Pryld-yield of below-ground biomass, PRL-pencil root length, PRW- pencil root width, ns- denotes presence and absence of significant difference at 5% level of probability

* and ** denotes significance at 5 and 1% level of probability.

The potential of vine production in *belg* season could be enhanced by using irrigation water and farmers that produce these vines could be encouraged by setting premium price to compensate for their investment in water during the relatively dry season.

### 3.4. Effects of medium of growth on growth and vine yield of sweet potatoes

#### 3.4.1. Vine length, branch number and leaf number

Significantly longer (P<0.05) vines were measured from plots treated with coffee husk and farmyard manure. The length of the vines was the shortest due to the treatment of soil medium with wood ash. The number of branches/plants increased as the crop stayed in the field. Significantly more (P<0.05) number of branches/plants were measured in the fourth month of the growth of vines from plots treated with compost and farmyard manure. The numbers of branches/plants were the least from the control plot. The number of leaves/plants did not show significant variations (P<0.05) in the second and third months. However, the number of leaves were significantly higher (P<0.05) due to the use of compost and coffee husk compared to the control. The numbers of leaves obtained due to the application of farmyard manure was intermediate (Fig 2 and Table 2). The absence of measurable variations in the second and third month unlike that of the fourth month could be due to the uniformity of the soil amendments in provision of weed suppression and moisture conservation in the aforementioned months. Conversely, the statistical difference measured on the vine, branch and leaf traits in the fourth month was due to the early mineralization of nutrients from farmyard manure and coffee husk compared to those in sawdust and wood ash.

**Table 2:** Length and number of sweet potato leaves, branches and vines as influenced by soil amendments over three month growth period.

Treatments	Vine length /branch (cm)			Branch number /plant (cm)			Leaf number /plant		
	two month	three month	four month	two month	three month	four month	two month	three month	four month
50% FYM	79.9	96.9	120.0	9.7	15.4	23.43	46.1	81.0	126.3
50% Saw dust	66.1	89.7	103.3	8.3	14.4	22.7	41.2	83.0	98.83
50% Wood ash	59.5	92.3	95.5	7.7	13.8	19.5	56.3	81.6	122.8
50% Compost	69.8	94.7	114.1	9.0	13.8	24.2	46.1	70.3	131.6
50% Coffee husk	65.6	100.8	129.1	9.4	14.7	14.7	43.1	78.8	138.6
100% Soil medium	65.7	91.3	111.8	9.4	14.8	14.4	51.9	90.0	118.8
LSD	ns	ns	22.4*	ns	ns	5.48*	ns	ns	14.5*
CV (%)	8.3	13.9	16.5	17.5	13.4	17.6	11.9	18.6	15.3

CV–coefficient of variation, LSD–least significant difference number of vines

* and ns- denoted presence and absence of significance difference at 5% level of probability, respectively. FYM was farmyard manure.

#### 3.4.2. Number of vines

**Fig 3:**
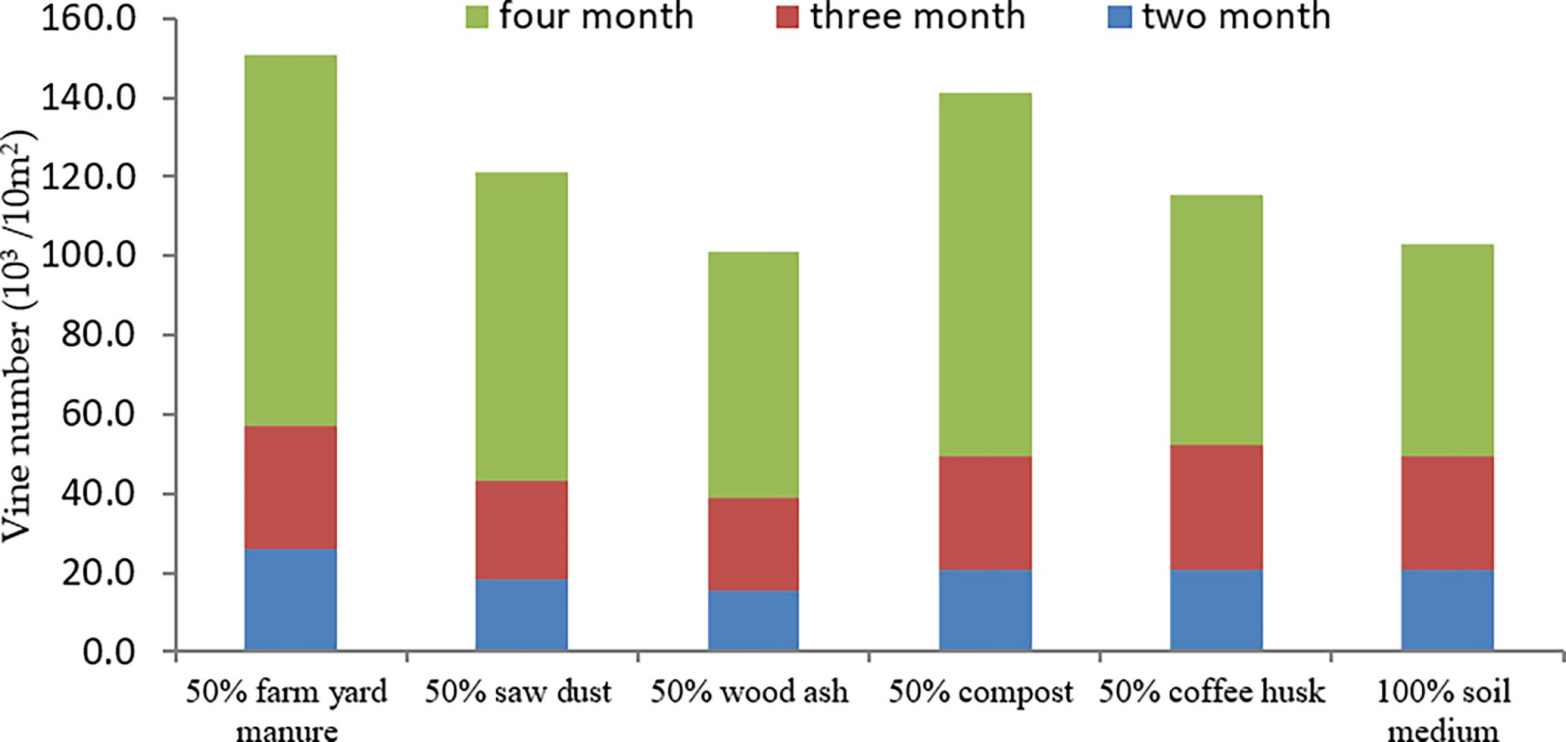
Number of sweet potato vines over four month period for variety *Kulfo*.

The number of vines harvested per plot was a function of the length of vines/branches, the number of branches/plants and the number of plants/plots. Considering the vine length of 30 cm for early generation seeds and assuming a plot size of 10 m^2^, the growth of vines was slow in the first two months, increased by two fold in the third month and reached its climax by doubling the figures in the fourth month in the *kulfo* variety. Results showed that a significantly higher number of vines were obtained from farmyard manure (93,720 vines / plot) and coffee husk (92,040.7 vines / plot) medium compared to the control (53,664 vines / plot) (Fig 3). The current finding of coffee husk was in conformity with Tenaw [26] who elaborated integrated use of coffee by-product and nitrogen fertilizer increased N uptake and water use of maize in Hawassa area, Southern Ethiopia. The superior performance of manure was also due to the timely nutrient release for the sweet potato plants [27].

#### 3.4.3. Internodes number and length

The number of internodes found in each vine did not differ statistically due to changes in soil amendments in this experiment. However, internodes length showed significant (P<0.05) variation due to differences in soil amendments (Table 3). Significantly (P<0.05) longer internodes were recorded due to farmyard manure (100.6% greater) compared to the control. The changes in length of internodes due to coffee husk and compost was 32.5% and 27.1%, respectively compared to the control. The increment in internodes length due to the use of wood ash and sawdust was higher than the control but lower than that of the farmyard manure, compost and coffee husk.

**Table 3:** Growth and biomass yield of sweet potatoes as influenced by soil amendments.

Treatments	NoI/ plant	LoI (cm)	% change	PRL (cm)	PRW (cm)	Byld(kg/ha)	% change	Pryld (kg/ha)	% change
50% Farmyard manure	149.2	3.33	100.6	10.04	2.88	51,166.6	62.0	4625.0	-7.5
50% Saw dust	147.1	1.86	12.0	9.58	3.77	27,250.0	-13.7	5083.3	1.7
50% Wood ash	150.3	1.83	10.2	10.38	3.39	36,000.0	13.9	5300.0	6.0
50% Compost	145.4	2.11	27.1	11.27	2.83	47,333.3	49.9	7383.3	47.7
50% Coffee husk	129.3	2.20	32.5	9.71	4.00	48,500.0	53.6	5625.0	12.5
100% Soil medium	139.9	1.66	0.0	8.25	3.66	31,583.3	0.00	5000.0	0.0
LSD	ns	1.12*		ns	ns	15712.3*		ns	
CV (%)	13.8	15.6		19.5	15.6	14.3		7.3	

NoI–Number of internodes, LoI–Length of internodes, PRL-pencil root length, PRW- pencil root width, Byld–yield of above-ground biomass, Pryld-yield of below-ground biomass

* and ns- denotes presence and absence of significance difference at 5% level of probability, respectively.

#### 3.4.4 Sweet potato vine yield and its components

**Fig 4:**
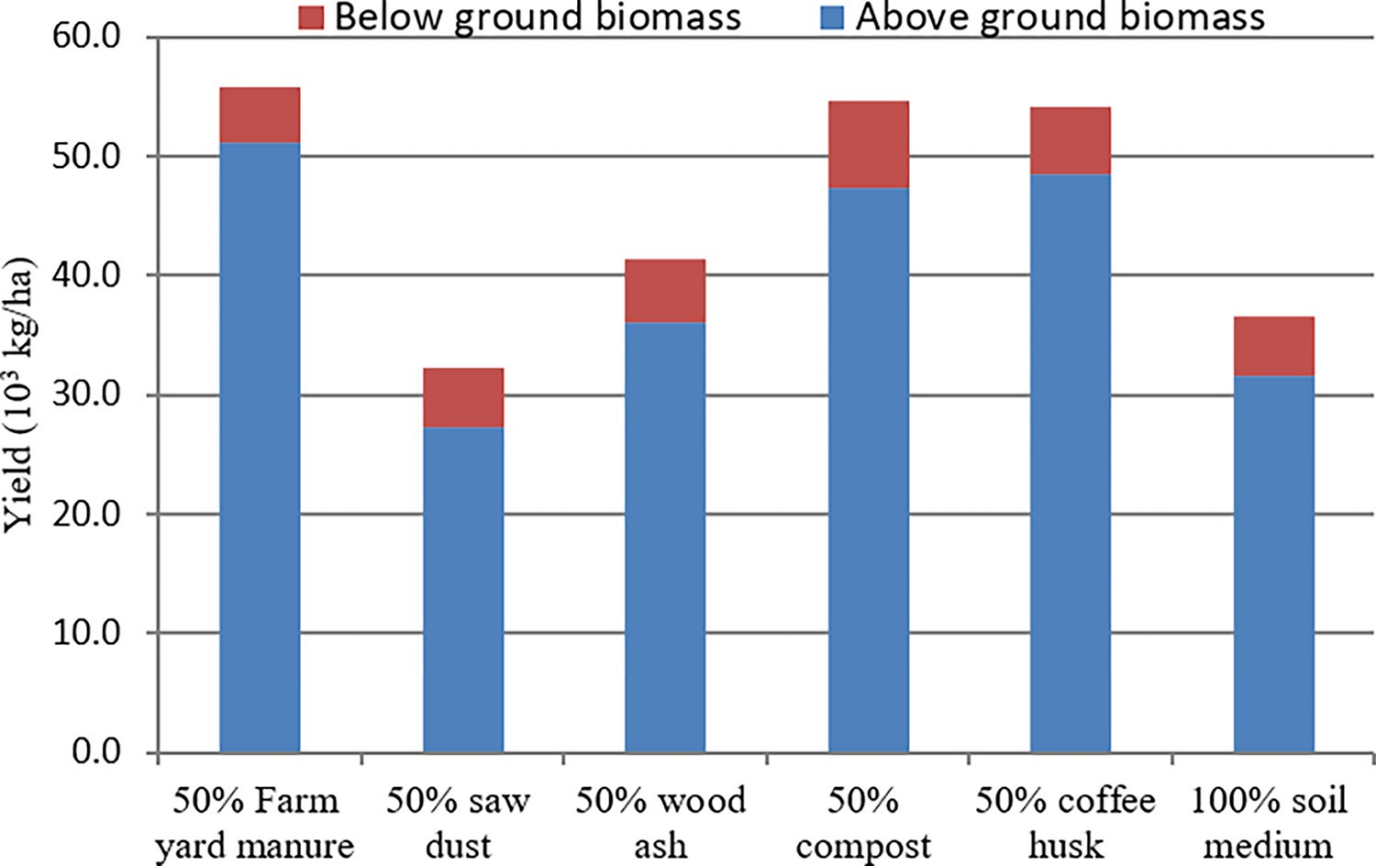
Proportion of above and below-ground biomass as affected by soil amendments.

Sweet potato roots produced in nursery beds were miniature roots regardless of the soil amendments, and hence called pencil roots despite growth for three to four months. The roots did form long but thin storage roots, and possess huge amount of adventitious lateral roots but did not develop fully because of the deliberate nutrition, temperatures and spacing factors that favored the growth of above-ground biomass to storage roots (below-ground biomass). Thus, the results showed that pencil root length, pencil root width, and below-ground biomass were not affected significantly (P<0.05) by the differences in soil amendments considered in this experiment (Table 3 and Fig 4). This result is against the findings of Sas-Paszt *et al*. [12] who compared different mulching materials and elaborated that only sawdust mulch significantly increased the number and size of apple fruit, attributing the response to the perennial nature of the test crop that would benefit from prolonged mineralization of the organic residue.

The vine yield and above-ground biomass differed significantly (P<0.05) due to the change in soil amendments (Table 3). Significantly higher above-ground biomass resulted due to the farmyard manure medium compared to the control. Results of above-ground biomass from the farmyard manure were comparable to the coffee husk and compost. The percentage increment in the above-ground biomass due to the farmyard manure, coffee husk and compost were 62.01%, 53.56% and 49.87% compared to the control. The alternative use of these three organic residues encouraged vine growth but suppressed root growth in sweet potato multiplication beds, which means that farmers might require artificial fertilizers for root growth and not vine production in the area. The increment in the vine yield or above-ground biomass was negative due to sawdust medium compared to the control, which could be due to late mineralization. This agreed with numerous authors who explored that the production of tubers was negatively correlated with that of the leaves and with the total aerial parts [28–30]. The reduction in the root yield and the increment in the production of vegetative parts were due to the decreasing distance between growing plants and the use of nitrogenous soil amendments [31,32]. The current finding agreed with Meniam *et al*. [9] who reported the use of farmyard manure or compost at the time of preparation of nursery beds.

### 3.5 Effects of *g*rowth medium x season interaction effect on branch number

Mean squares of season x growth medium interaction were significant on branch number measured after four months of sweet potato only (Annex table 1 in S1 File). Results showed that coffee husk and farmyard manure medium produced over 23 branches /plant during *meher* season whereas the control branch numbers of sweet potato measured after four months of sweet potato planting were as lowest number of branches, which is 14.4 branches /plant, were measured from control in the same season (Table 4). In the *belg* season, sweet potato plants maintained higher branches/plant (19.5) due to use of coffee husk compared to other treatments (Table 4). This could be mainly because coffee husk contained better nutrient release scheduling and soil moisture conserving attributes that benefitted the sweet potato crop. Coffee husk and farmyard manure amendments which produced higher branch numbers in both seasons also produced a higher vine yield, which depicts strong influence of branch numbers on vine yield in the sweet potato variety *Kulfo* multiplication beds.

**Table 4:** Numbers of branches of sweet potato measured after four months of sweet potato planting as affected by season x growth medium interaction.

Growth medium	*Meher*	*Belg*
50% FYM	23.4	14.8
50% Saw dust	22.7	13.0
50% Wood ash	19.6	13.8
50% Compost	24.3	13.2
50% Coffee husk	14.6	19.5
100% Soil medium	14.4	15.7
**LSD0.05**	**7.9**	

## 4. Conclusion and recommendations

The sweet potato vine business in Ethiopia is dominated by the informal sector; demand for quality planting materials of improved varieties is low and inconsistent. This was mainly due to the lack of demand for quality declared seed (QDS) from root producers, which was in turn due to the lack of awareness, low willingness to pay, and lack of registered QDS producers. Hence, Hawassa Agriculture Research Center was multiplying and delivering early-generation seeds of sweet potatoes to commercial vine multipliers. However, soils in Hawassa area lack many nutrients essential for plant growth. This resulted in complex signs and symptoms of nutritional disorders observed among growing sweet potato vines. To combat this constraint, there was a need to identify locally available soil amendments (farmyard manure, saw-dust, wood ash, and compost and coffee husk) for sweet potato vine production in net tunnels. Results showed that the use of each of these soil amendments improved the N content compared to the soil medium. Saw-dust produced superior N; coffee husk, compost and farmyard manure were intermediate and wood ash was the least as far as increment in N content was considered. Use of wood ash and farmyard manure increased the P content by 43% and 33.4%. Farmyard manure resulted in high EC, CEC, K and S but intermediate Zn content. Compost resulted in low pH, CEC and K but intermediate N, P and Ca. Pencil root length, pencil root width and pencil root yield were not significantly (P<0.05) different in the experiment. Coffee husk and farmyard manure medium produced higher number of branches (23 branches / plant) during *meher* whereas coffee husk produced the same during *belg* (14.4 branches / plant). Significantly higher above ground biomass (P<0.05) was obtained from farmyard manure, coffee husk and compost. Significantly taller (P<0.05) vines were measured from plots that received the coffee husk and farmyard manure. Significantly higher leaf number was recorded from plots that received the coffee husk, compost and farmyard manure. The largest branches were from plots that received the compost, farmyard manure and saw dust. The best internodes length was obtained from plots treated with the farmyard manure. It was obvious from the study that the practice of using wood ash and sawdust for sweet potatoes vine multiplication was not conducive as it was associated negatively with above ground biomass production and shortens the internodes length of the *Kulfo* variety. Farmyard manure increased vine yield by 62.01% whereas it decreased the root yield by 7.5%. Coffee husk increased the vine production by 53.56% whereas increased root yield by only 12.5%. This was mainly due to timely mineralization, enhanced nutrient uptake and moisture use. The current result encourages use of organic residues for small scale vine multiplication and significantly reduced cost for artificial fertilizers which otherwise could be required for commercial sweet potato vine multiplication. Hence, this finding showed that vine multipliers and sweet potato farmers can prepare rapid multiplication sweet potato beds to produce sufficient vines through use of either farmyard manure or coffee husk in *belg* and *meher* seasons in Hawassa and other areas that had similar agro-ecologies.

## Supporting information

S1 Table Row data. (XLSX)

S2 Table Climate data. (XLSX)

S1 File (DOCX)
