# Supplementary material for: Effects of soil amendments on growth and biomass yield of early generation seeds of sweet potato (Ipomoea batatas (L.) Lam) grown in net tunnels
Source: PLoS One. 2023 Nov 15;18(11):e0290585. doi: 10.1371/journal.pone.0290585 (PMC10651028; doi:10.1371/journal.pone.0290585)
Supplement: S1 File — (DOCX) [file pone.0290585.s003.docx]

Annex table 1. Analysis of variance (ANOVA) for growth and biomass of sweet potatoes grown in net tunnels under soil amendment experiment

| Source of variation | DF | VL2M | VL3M | VL4M | B2M | B3M | B4M | L2M | L3M |
| --- | --- | --- | --- | --- | --- | --- | --- | --- | --- |
| Season (S) | 1 | 1160* | 1590* | 1837* | 33.2ns | 23.6ns | 209.7** | 14228* | 72316*** |
| Error | 4 | 105 | 76 | 219 | 6.1 | 28.3 | 6.8 | 725 | 651 |
| Medium (M) | 5 | 279ns | 99* | 641ns | 3.5ns | 2.2* | 12.9ns | 191ns | 244* |
| S xM | 5 | 154 ns | 173 ns | 391 ns | 11.0ns | 41.4 ns | 63* | 251ns | 375ns |
| Error | 20 | 76 | 117 | 57 | 6.7 | 47.1 | 23.2 | 108 | 226 |
| CV(%) |  | 8.3 | 13.9 | 6.5 | 17.5 | 13.4 | 17.6 | 11.9 | 8.6 |

VL2M- vine length after two months, VL3M-vine length after three months, VL4M-vine length after four months, B2M – branch number after two months, B3M - branch number after three months, B4M - branch number after four months, L2M – leaf number after two months, L3M – leaf number after three months, L4M-leaf number after four months of sweet potato planting

Annex able 2. Analysis of variance (ANOVA) for growth and biomass of sweet potatoes grown in net tunnels under soil amendment experiment

| Source of variation | DF | L4M | Soil  moisture | NoI | LoI | PRL | PRW | Byld | Pryld | EC |
| --- | --- | --- | --- | --- | --- | --- | --- | --- | --- | --- |
| Season (s) | 1 | 58201*** | 84235** | 812ns | 1.6ns | 189* | 2815*** | 6806250000** | 85562500** | 0.000 |
| Error | 4 | 223 | 3844 | 689 | 0.89ns | 17.1 | 15 | 261034722 | 4201180 | 0.000 |
| Medium (m) | 5 | 222ns | 3874** | 370ns | 0.98ns | 5.9ns | 27.8ns | 599477777* | 5750111ns | 0.061*** |
| S x M | 5 | 385ns | 163ns | 0.803ns | 2.2ns | 7.8ns | 27.7ns | 25166666ns | 3261000ns | 0.000 |
| Error | 20 | 161 | 883 | 394 | 0.17 | 8.5 | 38.5 | 190976388 | 154115972 | 0.000 |
| CV(%) |  | 15.4 | 4.6 | 13.8 | 15.6 | 9.5 | 25.5 | 24.5 | 31.3 | 1.73 |

PRL-pencil root length, YAGB – Yield of above ground biomass, YBGB- yield of below ground biomass, NoI – number of internodes, LoI-length of internodes

Annex table 3. Soil micronutrients as affected by soil amendments at Hawassa *Chefe* site

| Treatments | Fe (mg  /kg) | Mn  (mg  /kg) | Zn (mg  /kg) | Mo (mg  /kg) | Co (mg  /kg) | Na (mg  /kg) | B (mg  /kg) | Cu (mg  /kg) | Fe (mg  /kg) | Al (mg  /kg) | Si (mg  /kg) |
| --- | --- | --- | --- | --- | --- | --- | --- | --- | --- | --- | --- |
| 50% Farmyard manure | 76.1 | 205.5 | 18.1 | 0.123 | 1.17 | 123.3 | 0.05 | 2.52 | 76.2 | 690.1 | 814.0 |
| 50% Saw dust | 31.8 | 266.5 | 14.4 | 0.130 | 1.21 | 107.5 | 0.32 | 2.22 | 76.1 | 755.6 | 857.0 |
| 50% Wood ash | 103 | 180.2 | 23.4 | 0.133 | 1.05 | 256.9 | 0.65 | 2.82 | 104.0 | 702.1 | 917.0 |
| 50% Compost | 87.1 | 198.2 | 15.1 | 0.137 | 1.11 | 84.7 | 0.05 | 2.15 | 102.7 | 788.7 | 845.6 |
| 50% Coffee husk | 78.4 | 210.2 | 16.4 | 0.123 | 1.15 | 65.2 | 0.05 | 2.46 | 79.5 | 725.1 | 804.3 |
| 100% Soil medium | 75.1 | 421.4 | 14.5 | 0.133 | 1.13 | 71.6 | 0.05 | 1.84 | 73.9 | 795.5 | 874.0 |
| **LSD** | **34.6**** | **2.52**** | **0.38**** | **ns** | **0.017**** | **2.49**** | **ns** | **0.17**** | **0.87**** | **7.45**** | **14.6**** |
| **CV (%)** | **19.2** | **0.90** | **1.98** | **3.72** | **0.97** | **1.31** | **16.3** | **4.6** | **0.64** | **0.62** | **1.07** |

Annex table 4. Nitrogen, Phosphorus, sulfur, pH and moisture of soils as affected by soil amendments

| Treatments | pH  H_2_0 | % change | Soilmoisture (%) | % change | N  (%) | % change | P(mg  /kg) | % change | S  (mg/kg) | % change |
| --- | --- | --- | --- | --- | --- | --- | --- | --- | --- | --- |
| 50% marmyardmanure | 7.33 | -0.9 | 217.0 | 17.3 | 0.19 | 58.3 | 535.1 | 33.4 | 25.8 | 59.3 |
| 50% Saw dust | 7.50 | 1.4 | 237.5 | 28.4 | 0.45 | 275 | 339.6 | -15.3 | 13.80 | -14.8 |
| 50% Wood ash | 8.06 | 8.9 | 218.8 | 18.3 | 0.13 | 8.3 | 573.6 | 43.0 | 39.9 | 146.3 |
| 50% Compost | 6.71 | -9.3 | 190.6 | 3.0 | 0.22 | 83.3 | 421.3 | 5.0 | 22.8 | 40.7 |
| 50% Coffee husk | 6.96 | -5.9 | 169.6 | -8.3 | 0.24 | 100 | 389.0 | -3.0 | 20.3 | 25.3 |
| 100% Soil medium | 7.40 | 0.0 | 185.0 | 0.0 | 0.12 | 0.0 | 401.1 | 0.0 | 16.2 | 0.0 |
| LSD | 0.201** |  | 33.8* |  | 0.11** |  | 4.59** |  | 0.67** |  |
| CV (%) | 2.3 |  | 14.6 |  | 1.3 |  | 0.92 |  | 2.53 |  |

Annex table 5. EC, CEC, potassium, calcium and magnesium of soils as affected by soil amendments,

| Treatments | EC (mS  /cm) | % change | CEC (Meq/  100g soil) | % change | K  (mg  /kg) | % change | Ca  (mg  /kg) | % change | Mg  (mg  /kg) | % change |
| --- | --- | --- | --- | --- | --- | --- | --- | --- | --- | --- |
| 50% Farmyard manure | 0.32 | 146.2 | 24.08 | 26.5 | 2709.6 | 45.5 | 3410.3 | -3.6 | 558.1 | 32.5 |
| 50% Saw dust | 0.10 | -23.1 | 24.81 | 30.3 | 1872.6 | 0.6 | 3567.6 | 0.9 | 431.0 | 2.3 |
| 50% Wood ash | 0.23 | 76.9 | 24.98 | 31.2 | 2738.3 | 47.0 | 4207.3 | 18.9 | 887.3 | 110.6 |
| 50% Compost | 0.17 | 30.8 | 19.07 | 0.2 | 1751.6 | -5.9 | 3681.0 | 4.1 | 425.3 | 0.9 |
| 50% Coffee husk | 0.35 | 169.2 | 27.99 | 47.0 | 2530.0 | 35.9 | 3527.3 | -0.3 | 463.3 | 10.0 |
| 100% Soil medium | 0.13 | 0.0 | 19.04 | 0.0 | 1862.3 | 0.0 | 3537.3 | 0.0 | 421.3 | 0.0 |
| **LSD** | **0.104**** |  | **0.095**** |  | **33.4**** |  | **44.04**** |  | **3.37**** |  |
| **CV (%)** | **1.72** |  | **0.36** |  | **0.95** |  | **0.75** |  | **0.56** |  |

Annex Table 6. Rating soil fertility parameters adopted for this study

| **No** | **Soil parameter** | **Status** | **Critical level** | **Citation** | **No** | **Soil parameter** | **Status** | **Critical level** | **Citation** |
| --- | --- | --- | --- | --- | --- | --- | --- | --- | --- |
| 1 | Soil pH  (water) | Strongly acidic  Moderately acidic  Neutral  Moderately alkaline  Strongly alkaline | <5.5 5.6-6.5  6.6-7.3  7.3-8.4  >8.4 | Ethiosis Team Analysis (2014) and Brady(1985) | 9 | Ca  (mg/ka or ppm) | Very low  Low  Moderate High Very high | ,<,2  2 - 5  5 - 10  10 - 20  .>20 | Hazelton and Murphy (2007)  (FAO, 2006) |
| 2 | EC (mS/cm or ds/m) | Salt free  Very slightly saline  Slightly saline  Moderately saline  Strongly saline | <2  2-4  4-8  8-16  >16 | Ethiosis Team Analysis (2014) and Muhr et al. (1963) | 10 | Mg  (mg/ka or ppm) | Very low  Low  Moderate High Very high | <0.3  0.3 - 1  1 - 3  3 - 8  >8 | Hazelton and Murphy (2007)  (FAO, 2006) |
| 3 | CEC (me/100 soil g) | Indicate soil infertility  Minimum value  Optimum  High  Very high | <4  5-15  15-25  25-40  >40 | Landon (1984) | 11 | Available K (mg/kg) | Very low  Low  Moderate High Very high | <0.2  0.2 – 0.3  0.3 – 0.6  0.6 – 1.2  >1.2 | Hazelton and Murphy (2007)  (FAO, 2006) |
| 4 | Available P (mg/kg) | Very low  Low  Optimum  High  Very high | 0-15  15-30  30-80  80-150  >150 | Ethiosis Team Analysis (2014), and Muhr et al. (1963). | 12 | Zn  (mg/ka or ppm) | Deficient  Marginal  Sufficient I  Sufficient II | <0.6 0.6-1.2  1.2-2.4  >2.4 | Kirmani et al. (2011) |
| 5 | Nitrogen (%) | Very low  Low  Optimum  High  Very High | <0.15  0.15-0.3  0.3-0.55  0.55-1.05  >1.05 | Ethiosis Team Analysis (2014), Subbiah and Asija (1956) | 13 | Cu  (mg/ka or ppm) | Deficient  Marginal  Sufficient I  Sufficient II  Sufficient III | <0.2 0.2- 0.4  0.4-0.8  0.8-1.6  1.6-3.2 | Lindsay and Novell (1978) and Kirmani et al. (2011) |
| 6 | OC% | Low  Medium  High | <2%  2-10%  >10% | Landon (1984) and Ethiosis Team Analysis (2014) | 14 | Fe (mg/ka or ppm) | Deficient  Marginal  Sufficient I  Sufficient II  Sufficient III | <4.5  4.5-9  9-18  18-27  >27 | Lindsay and Novell (1978) |
| 7 | Organic matter (%) | Very low  Low  Optimum  High  Very high | < 2  2.0-3.0  3.0-7.0  7.0-8.0 >8.0 | Ethiosis Team Analysis (2014)  (FAO, 2006) | 15 | Mn  (mg/kg or ppm) | Deficient  Marginal  Sufficient I  Sufficient II | <2.5  2.5-3.5  3.5-7  >7 | Lindsay and Novell (1978) |
| 8 | Exchangeable K (Cmolc/kg) | Very low  Low  Optimum  High  Very high | <0.90  0.90-19  19-60  60-90  >90 | Ethiosis Team Analysis (2014) | 16 | Bulk density(g/cm3) | \|  \| \| --- \|   Very low  Low  Moderate High Very high | < 1.0  1.0-1.3  1.3-1.6  1.6-1.9  >1.9 | Hazelton and Murphy (2007) |
