# Supplementary material for: Effects of soil amendments on growth and biomass yield of early generation seeds of sweet potato (Ipomoea batatas (L.) Lam) grown in net tunnels
Source: PLoS One. 2023 Nov 15;18(11):e0290585. doi: 10.1371/journal.pone.0290585 (PMC10651028; doi:10.1371/journal.pone.0290585)
Supplement: Attachment — Submitted filename: Response to reviewer.docx [file pone.0290585.s004.docx]

Response to reviewer #1

| # | Question | Response |
| --- | --- | --- |
| 1 | Correct misspelled words | farm yard written as farmyard throughout the text  sweetpotato written as sweet potato >> >> micro nutrients written as micronutrients >> >> by products hyphenated as by-products >> >> Ssweetpotato written as sweet potato >> >> |
| 2 | Clarify the type of ANOVA performed. Given the experimental design (treatment x season x year) | The two way analysis of variance was done for season x medium of growth because the homogeneity test showed equal variances across years as shown between lines 161-162 and 216-217 |
| 3 | Mean square is not the final step of an ANOVA. F-statistic is which is MS treatment / MS residual | The F-test was conducted as post hoc test and results were presented as mean separation using LSD as shown between lines 164- 165 |
| 4 | Show interpretation for a significant 2-way interaction (season x medium of growth) | An interpretation was given for two way ANOVA between lines 364 - 362  However, where interaction was not significant main effects were discussed thoroughly |
| 5 | What are “rapid multiplication beds”? | This has been explained between lines 12-13 |
| 6 | Clarify that the planting material needed is to be disease free | Has been clarified in line 65 |
| 7 | The composition of soil medium would be half and that of added medium would be half”; do you mean 50:50 v/v (volume to volume)? | Has been shown in line 112 |
| 8 | Clarify the range of values considered ‘superior’? ‘high’? | Has been rated as shown in Annex table 6 between lines 227-242 and 240-242. References were given between lines reference number 23 to 32 |
| 9 | Intern? | Changed to in turn in lines 62, 188 and 137 |
| 10 | Poor plant performance? | Has been described properly as low vine yield |
| 11 | Superior N | Has been described by rating of nitrogen between lines 256 - 258 |
| 12 | It is difficult to understand a sentence such as “However, effects of soil amendments were significant on soil moisture (P<0.01), YAGB (P<0.01), VLThM (P<0.05), BNThM (P<0.05), LNThM (P<0.05) and LoI (P<0.05) (Annex table 1 and 2).” Perhaps you can simply refer to Table 1 rather than write this detail in the text. What do the abbreviations stand for; consider replacing with more common words | Has been written as suggested by reviewers between lines 168 - 180 |
| 13 | Rather than list percentages of various nutrients and soil properties in the text, consider generating a bar chart to make comparisons easier among treatments. | This was fixed as percentage because percentage shows better the increase soil physico-chemical properties due to treatment effect |
| 14 | Table 3/4. It would be easier to visualize these patterns through time as a graph rather than a table | Line graphs show dry matter accumulation over months but wouldn’t show a sequential increment of growth components against the growth medium considered as a treatment. Moreover it’s stack. |
| 15 |  |  |

|  | 50% Farmyard manure | 50% Saw dust | 50% Wood ash | 50% Compost | 50% Coffee husk | 100% Soil medium |
| --- | --- | --- | --- | --- | --- | --- |
| 2months | 25.8 | 18.3 | 15.3 | 20.9 | 20.6 | 20.6 |
| 3months | 31.3 | 24.8 | 23.7 | 28.4 | 31.6 | 28.6 |
| 4months | 93.7 | 78.2 | 62.1 | 92 | 63.3 | 53.7 |

Response to Reviewer #2

| # | Questions | Response |
| --- | --- | --- |
| 1 | Italicize scientific names | Has been corrected in line 1 and 27 as suggested by the reviewer |
| 2 | Design lacks clarity | Has been clarified between lines 15-17 |
| 3 | Farm yard manure is not written correctly | Has been written as farmyard manure throughout the text |
| 4 | Clarify sentence in line 24 | Has been correctly written in line 28 |
| 5 | Rephrase line 27 | Has been rephrased in line 20 |
| 6 | Verb change suggested | Verb change accepted |
| 7 | Measurement clarity between lines 9 to 100 | Has been corrected between lines 111 to 112 |
| 8 | The basis for 30 cm in the formula | A vine is considered a good planting material with minimum length of 30 cm and that has been fixed in the formula |
| 9 | Explain pencil roots between lines 138 - 139 | Has been briefly explained between lines 150 - 151 |
| 10 | Put rainfall in SI units | Rainfall has been labeled in millimeters |
| 11 | More branchy should be changed | Change to had more branch numbers in line 240 |
| 12 | Correct heading for the table in line 189 | The heading was correctly given in line 258 |
| 13 | Correct AI in line 211 | Has been made Al in line 190 |
| 14 | <<Compared with>> need change in line 245 | Changed to << compared to>> throughout the text |
| 15 | In line 256, change the table heading | Has been changed in line 286 and 315 |
| 16 | Clarify units of the figure | The figure measures vine numbers and hence the naming corrected as suggested by the reviewer |
| 17 | Farm yard <<medium>> to change in line 276 | In line 310, medium change to manure |
| 18 | Slightly higher in line 281 | Changed to higher than in line 313 |
| 19 | In line 283, the change percentage was not clear | The change percentage was calculated from the control as shown in line 154 using equation 3 |
| 20 | Line 290 spelling correction suggested | Has been corrected in line 320 |
| 21 | In Line 313, figure heading was suggested for correction | Has been corrected in 348 as suggested by the reviewer |
| 22 | Mean squares to be removed | Change to ANOVA table as suggested |
| 24 | Change of table headings suggested in line 423, 427 and 429 | Has been corrected in lines 519, 523 and 525 |
