# Supplementary material for: Effects of soil amendments on growth and biomass yield of early generation seeds of sweet potato (Ipomoea batatas (L.) Lam) grown in net tunnels
Source: PLoS One. 2023 Nov 15;18(11):e0290585. doi: 10.1371/journal.pone.0290585 (PMC10651028; doi:10.1371/journal.pone.0290585)
Supplement: Peer review history

# Author comment: Author response to previous submission

**DOI:** 10.1371/journal.pone.0290585.r001

1 Aug 2023

Attachment Submitted filename: Response to reviewer.docx Click here for additional data file.

# Editor report: Decision Letter 0

**DOI:** 10.1371/journal.pone.0290585.r002

**Authors:** Marcela Pagano

11 Aug 2023

Effects of soil amendments on growth and biomass yield of early generation seeds of sweet potato (Ipomoea batatas (L.) Lam) grown in net tunnels

PONE-D-23-23926

Dear Dr. Markos,

We’re pleased to inform you that your manuscript has been judged scientifically suitable for publication and will be formally accepted for publication once it meets all outstanding technical requirements.

Within one week, you’ll receive an e-mail detailing the required amendments. When these have been addressed, you’ll receive a formal acceptance letter and your manuscript will be scheduled for publication.

An invoice for payment will follow shortly after the formal acceptance. To ensure an efficient process, please log into Editorial Manager at http://www.editorialmanager.com/pone/, click the 'Update My Information' link at the top of the page, and double check that your user information is up-to-date. If you have any billing related questions, please contact our Author Billing department directly at authorbilling@plos.org.

If your institution or institutions have a press office, please notify them about your upcoming paper to help maximize its impact. If they’ll be preparing press materials, please inform our press team as soon as possible -- no later than 48 hours after receiving the formal acceptance. Your manuscript will remain under strict press embargo until 2 pm Eastern Time on the date of publication. For more information, please contact onepress@plos.org.

Kind regards,

Marcela Pagano, Ph.D, M.D.

Academic Editor

PLOS ONE

Journal Requirements:

1. In your Methods section, please provide additional information regarding the permits you obtained for the work. Please ensure you have included the full name of the authority that approved the field site access and, if no permits were required, a brief statement explaining why.

2. Thank you for stating the following financial disclosure: 

"Authors received funds for this research from Southern Agriculture Research Institute"

Please state what role the funders took in the study.  If the funders had no role, please state: "The funders had no role in study design, data collection and analysis, decision to publish, or preparation of the manuscript." 

If this statement is not correct you must amend it as needed. 

Please respond by return e-mail so that we can amend your financial disclosure and competing interests on your behalf.

3. Please include your full ethics statement in the ‘Methods’ section of your manuscript file. In your statement, please include the full name of the IRB or ethics committee who approved or waived your study, as well as whether or not you obtained informed written or verbal consent. If consent was waived for your study, please include this information in your statement as well. 

# Editor report: Acceptance letter

**DOI:** 10.1371/journal.pone.0290585.r003

**Authors:** Marcela Pagano

23 Aug 2023

PONE-D-23-23926 

Effects of soil amendments on growth and biomass yield of early generation seeds of sweet potato (*Ipomoea batatas* (L.) Lam) grown in net tunnels 

Dear Dr. Markos:

I'm pleased to inform you that your manuscript has been deemed suitable for publication in PLOS ONE. Congratulations! Your manuscript is now with our production department. 

If your institution or institutions have a press office, please let them know about your upcoming paper now to help maximize its impact. If they'll be preparing press materials, please inform our press team within the next 48 hours. Your manuscript will remain under strict press embargo until 2 pm Eastern Time on the date of publication. For more information please contact onepress@plos.org.

If we can help with anything else, please email us at plosone@plos.org. 

Thank you for submitting your work to PLOS ONE and supporting open access. 

Kind regards, 

PLOS ONE Editorial Office Staff

on behalf of

Dr. Marcela Pagano 

Academic Editor

PLOS ONE
